# The effect of chronic cerebral hypoperfusion on the pathology of Alzheimer's disease: A positron emission tomography study in rats

**DOI:** 10.1038/s41598-019-50681-4

**Published:** 2019-10-01

**Authors:** Jae-Hyung Park, Jeong-Ho Hong, Sang-Woo Lee, Hyun Dong Ji, Jung-Ah Jung, Kyung-Wha Yoon, Jung-In Lee, Kyoung Sook Won, Bong-Il Song, Hae Won Kim

**Affiliations:** 1 Department of Physiology, School of Medicine, Keimyung University, Daegu, Republic of Korea; 2 Department of Neurology, School of Medicine, Keimyung University, Daegu, Republic of Korea; 3 Department of Nuclear Medicine, School of Medicine, Kyungpook National University, Daegu, Republic of Korea; 4 Department of Nuclear Medicine, School of Medicine, Keimyung University, Daegu, Republic of Korea

**Keywords:** Cognitive control, Neuro-vascular interactions

## Abstract

Cerebrovascular disease is a potential risk factor for Alzheimer's disease (AD). Although acute cerebral hypoperfusion causes neuronal necrosis and infarction, chronic cerebral hypoperfusion induces apoptosis in neurons, but its effects on the cognitive impairment are not clear. The purpose of this study was to evaluate the effects of chronic cerebral hypoperfusion on AD pathology and cerebral glucose metabolism. A model of chronic cerebral hypoperfusion was established by ligating the common carotid arteries bilaterally in adult male rats (CAL group). Sham-operated rats underwent the same procedures without artery ligation (control group). At 12 weeks after ligation, expression levels of amyloid-β (Aβ) and hyperphosphorylated tau (p-tau), as well as the regional cerebral glucose metabolism, were evaluated using Western blots and positron emission tomography with fluorine-18 fluorodeoxyglucose, respectively. The expression levels of Aβ in the frontal cortex and hippocampus and of p-tau in the temporal cortex were significantly higher in the CAL group than those in the control group. The cerebral glucose metabolism of the amygdala, entorhinal cortex, and hippocampus was significantly decreased in the CAL group compared to that in the control. These results suggest that chronic cerebral hypoperfusion can induce AD pathology and may play a significant role in AD development.

## Introduction

Alzheimer’s disease (AD) is a neurodegenerative disease and the most common cause of dementia worldwide. The global prevalence of AD was estimated to be 3.9% in the elderly^1^. Previous epidemiological, biochemical, genetic, and animal studies have suggested various explanations regarding the etiology of AD. According to the amyloid-β (Aβ) cascade hypothesis, the accumulation of cerebral Aβ peptides is the initial pathological process during AD, which leads to the formation of extracellular senile plaques^2^. Additionally, AD pathology is characterized by intracellular neurofibrillary tangles formed by the microtubule-associated protein tau;^3^ however, a clear pathophysiology for AD remains to be confirmed^4^.

Recent studies have shown that AD, like other chronic diseases, develops as a result of multiple factors and is not the result of solely one factor. Among these factors, cerebrovascular disease has once again gained attention for its important role in AD development^5,6^. A higher incidence of cerebrovascular disease has been reported in AD patients than in age-matched controls in clinical studies^7^. The prevalence of vascular pathology ranges from 8% to 35% according to an autopsy series of patients with AD^8^. A population-based study evaluated the effects of modifiable risk factors on cognitive decline and dementia and found sufficiently strong evidence that the management of vascular risk factors (e.g., diabetes, obesity, smoking, high cholesterol, and hypertension) could reduce the risk of dementia and prevent the development of AD^6^.

The important role of chronic cerebral hypoperfusion in dementia has already emerged at the front edge of neurology research. While severe cerebral hypoperfusion leads within approximately 3 hours to an acute infarction through necrosis of neuronal cells^9^, chronic cerebral hypoperfusion of a lesser degree is known to cause neurodegeneration over a period of months to years through neuronal apoptosis without acute infarction^10^, and individuals with chronic cerebral hypoperfusion usually have cognitive deficits to various degrees^11^. Previous *in vivo* studies using animal models of chronic cerebral hypoperfusion revealed that chronic ischemia contributes to AD development with increases in cerebral Aβ burden and hyperphosphorylated tau (p-tau) levels^12,13^. Clinically, chronic cerebral hypoperfusion has been known to present as white matter lesions on magnetic resonance imaging (MRI), and our study group reported that white matter lesions are associated with increased cerebral Aβ burden in patients with cognitive impairment^14^. Although a number of studies have established the effect of cerebral large vessel disease on multi-infarct dementia^6^, the effect of the more frequent consequences of chronic cerebral hypoperfusion on AD pathogenesis remains debatable. Furthermore, the effects of chronic cerebral hypoperfusion on the neuronal activity of the whole brain have not been evaluated yet. Positron emission tomography (PET) is known to be a suitable tool for identifying AD pathology and for assessing AD progression. Furthermore, F-18 fluorodeoxyglucose (FDG) PET can be used to measure the regional cerebral glucose metabolism of the whole brain, which reflects neuronal activity^15^. Thus, the purpose of this study was to evaluate the effects of chronic cerebral hypoperfusion on AD pathology and cerebral glucose metabolism in rats using F-18 FDG PET.

## Results

### AD pathology

**Figure 1:**
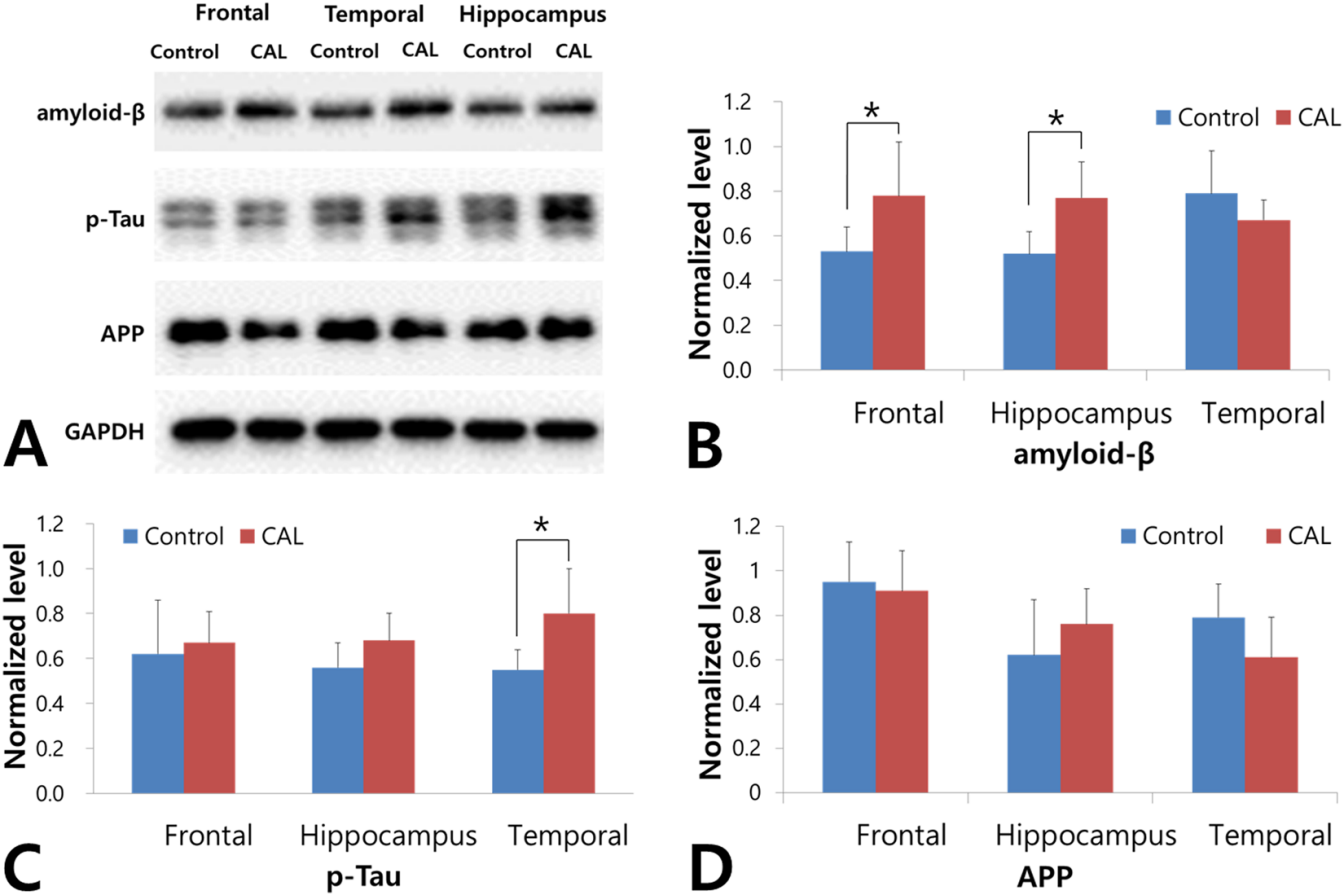
Comparison of AD pathology between the common carotid artery ligation (CAL) and the control groups. **(A)** The expression levels of amyloid-β (Aβ), hyperphosphorylated tau (p-tau), and amyloid precursor protein (APP) were evaluated at 12 weeks after bilateral CAL using western blot analysis. **(B)** The Aβ levels in the frontal cortex and hippocampus are significantly higher in the CAL group than those in the control group. **(C)** The expression of p-tau in the temporal cortex of the CAL group is significantly higher than that in the control group. **(D)** There are no significant differences in APP expression between the control and the CAL group. Asterisks indicate statistical significance: ^*^p < 0.05.

The Aβ levels of the frontal cortex and hippocampus in the group with bilateral common carotid artery ligation (CAL) were significantly higher than those in the control group (0.53 ± 0.11 vs. 0.77 ± 0.26, *p* = 0.034 and 0.52 ± 0.10 vs. 0.79 ± 0.19, *p* = 0.001, respectively; Fig. 1A,B). The expression of Aβ in the temporal cortex was not significantly different between the CAL group and the control group (0.79 ± 0.19 vs. 0.69 ± 0.11, *p* = 0.243). By contrast, the expression of p-tau in the temporal cortex of the CAL group was significantly elevated in comparison to that of the control group (0.55 ± 0.09 vs. 0.86 ± 0.32, *p* = 0.018; Fig. 1C). The expression levels of p-tau in the frontal cortex and hippocampus were not significantly different between the CAL and the control groups (0.62 ± 0.24 vs. 0.68 ± 0.34, *p* = 0.309 and 0.56 ± 0.11 vs. 0.67 ± 0.11, *p* = 0.078, respectively). No significant differences in the amyloid precursor protein (APP) between the control and the CAL group were observed (0.95 ± 0.18 vs. 0.93 ± 0.28, *p* = 0.651; 0.62 ± 0.25 vs. 0.75 ± 0.26, *p* = 0.087; and 0.79 ± 0.15 vs. 0.65 ± 0.15, *p* = 0.213 for the frontal, hippocampal, and temporal region, respectively; Fig. 1D).

### Cerebral glucose metabolism

**Figure 2:**
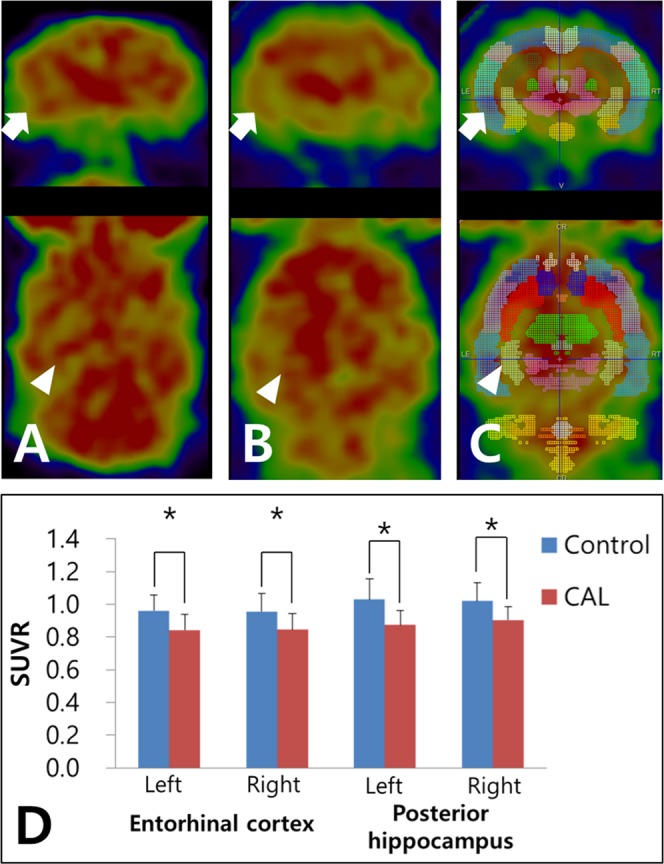
Cerebral glucose metabolism measured by F-18 FDG PET. (**A**) A sham-operated rat (control group) showing no abnormal glucose metabolism of the entorhinal cortex (arrow) and posterior hippocampus (arrowhead). **(B)** A rat with bilateral common carotid artery ligation (CAL group) showing a decreased glucose metabolism of the entorhinal cortex and posterior hippocampus. **(C)** Regional standardized F-18 FDG uptake values (SUVRs) obtained from the W. Schiffer rat brain volume-of-interests using the PMOD software package (see Methods). **(D)** The regional SUVR of the left entorhinal cortex and bilateral posterior hippocampus in the CAL group are significantly lower than those in the control group. Asterisks indicate statistical significance: ^*^p < 0.05.

For the semiquantitative analysis of F-18 FDG brain PET, the regional standardized F-18 FDG uptake value ratios were calculated with the cerebellum (SUVR_cbl_) and whole brain (SUVR_WB_) as reference tissues. The SUVR_cbl_ of the bilateral amygdala, bilateral entorhinal cortex, right anterodorsal hippocampus, and bilateral posterior hippocampus were significantly lower in the CAL group than those in the control group (*p* = 0.002, *p* = 0.028, *p* = 0.005, *p* = 0.028, *p* = 0.036, *p* = 0.021, and *p* = 0.026, respectively; Fig. 2). There were no significant SUVR_cbl_ differences in other regions of the brain (Table 1). Also, with SUVR_WB_ comparison, the SUVR_WB_ of the left amygdala, left entorhinal cortex, right frontal association cortex, and left posterior hippocampus were significantly lower in the CAL group than those in the control group (*p* = 0.021, *p* = 0.036, *p* = 0.016, and *p* = 0.043, respectively; Supplementary Table 1).

**Table 1:** Comparisons of regional standardized uptake value ratios^*^ between the control and the bilateral common carotid artery ligation (CAL) group.

Regions	Side	Control group	CAL group	*p*-value
Accumbens	Lt	1.07 (0.11)	0.96 (0.12)	0.062
	Rt	1.07 (0.09)	1.00 (0.10)	0.118
Amygdala	Lt	0.94 (0.07)	0.78 (0.04)	0.002
	Rt	0.91 (0.10)	0.81 (0.05)	0.028
Auditory cortex	Lt	0.94 (0.01)	0.91 (0.06)	0.394
	Rt	0.90 (0.09)	0.87 (0.05)	0.350
Cingulate cortex	Lt	1.07 (0.18)	1.01 (0.05)	0.445
	Rt	1.06 (0.22)	0.95 (0.04)	0.220
Entorhinal cortex	Lt	0.96 (0.10)	0.83 (0.05)	0.005
	Rt	0.96 (0.11)	0.84 (0.07)	0.028
Frontal association cortex	Lt	0.95 (0.10)	0.87 (0.05)	0.084
	Rt	0.91 (0.08)	0.89 (0.04)	0.596
Insular cortex	Lt	1.04 (0.13)	0.93 (0.12)	0.078
	Rt	1.02 (0.09)	0.97 (0.10)	0.219
Medial prefrontal cortex	Lt	1.18 (0.18)	1.05 (0.10)	0.116
	Rt	1.19 (0.17)	1.05 (0.08)	0.069
Motor cortex	Lt	0.94 (0.12)	0.91 (0.17)	0.513
	Rt	0.93 (0.13)	0.87 (0.08)	0.259
Orbitofrontal cortex	Lt	1.07 (0.13)	0.97 (0.08)	0.076
	Rt	1.09 (0.09)	1.01 (0.08)	0.088
Para cortex	Lt	0.85 (0.12)	0.83 (0.05)	0.751
	Rt	0.85 (0.13)	0.82 (0.04)	0.472
Retrosplenial cortex	Lt	0.91 (0.09)	0.89 (0.06)	0.623
	Rt	0.94 (0.14)	0.89 (0.06)	0.431
Somatosensory cortex	Lt	0.95 (0.11)	0.9 (0.04)	0.354
	Rt	0.93 (0.11)	0.89 (0.06)	0.379
Visual cortex	Lt	0.86 (0.11)	0.83 (0.06)	0.440
	Rt	0.86 (0.10)	0.81 (0.05)	0.278
Hippocampus anterodorsal	Lt	1.07 (0.12)	0.97 (0.06)	0.066
	Rt	1.08 (0.12)	0.96 (0.07)	0.036
Hippocampus posterior	Lt	1.03 (0.13)	0.89 (0.09)	0.021
	Rt	1.02 (0.11)	0.90 (0.07)	0.026

^*^Regional standardized uptake value ratio (SUVR_WB_) was calculated by dividing the standardized uptake value for each regional VOI by the standardized uptake value for the cerebellum as a reference region. All values are presented as mean (standard deviation).

### Recognition memory

The novel object recognition test was performed at 28 days after the bilateral CAL surgery to assess whether recognition memory is impaired by chronic cerebral hypoperfusion. In the short term memory retention test, the recognition index in the CAL group was significantly lower than that in the control group (0.53 ± 0.14 vs. 0.72 ± 0.11, *p* = 0.007). Also, in the long term memory retention test, the recognition index in the CAL group was significantly lower than that in the control group (0.54 ± 0.11 vs. 0.65 ± 0.09, *p* = 0.041).

### Cerebral blood flow

**Figure 3:**
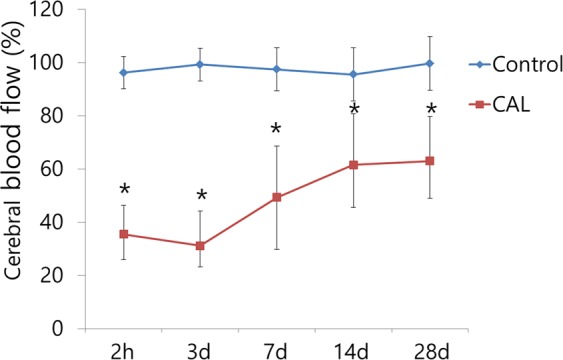
The cerebral blood flow (CBF) was measured at 2 hours and 3, 7, 14, and 28 days after the bilateral common carotid artery ligation (CAL) surgery using laser-Doppler flowmetry. At 2 hours and 3 days after the surgery, there was a significant decrease in the CBF values in the CAL group compared with those in the control group. On day 7, the CBF values in the CAL group began to recover but remained significantly lower until day 28, as compared with those in the control group. Asterisks indicate statistical significance: ^*^p < 0.05.

Using laser-Doppler flowmetry, the cerebral blood flow (CBF) was measured just before and at 2 hours and 3, 7, 14, and 28 days after the bilateral CAL surgery (Fig. 3). At 2 hours and 3 days after the surgery, there was a significant decrease in the CBF values in the CAL group compared with those in the control group (35.5 ± 10.7% vs. 96.1 ± 6.1%, *p* < 0.001 and 31.1 ± 13.0% vs. 99.1 ± 8.9%, *p* < 0.001). On day 7, the CBF values in the CAL group began to recover but remained significantly lower, as compared with those in the control group (49.3. ± 19.4% vs. 97.4 ± 8.1%, *p* < 0.001). The CBF values in the CAL group were still decreased significantly compared with those in the control group at 14 (61.5 ± 19.1% vs. 95.5 ± 9.9%, *p* = 0.001) and 28 days (62.9 ± 16.7% vs. 99.6 ± 10.0%, *p* < 0.001).

### Cerebral infarction

**Figure 4:**
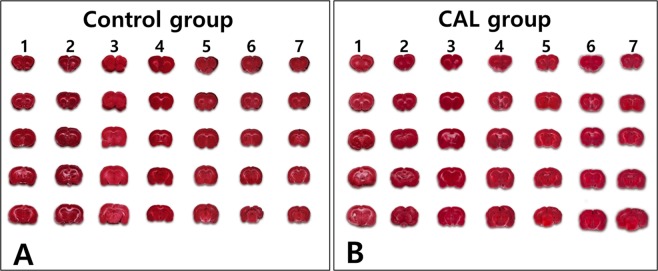
Cerebral infarct volume evaluated by the 2,3,5-triphenyltetrazolium chloride (TTC) assay (**A**) TTC assay showing that none of the seven rats in the control group exhibited cerebral infarction. **(B)** In the common carotid artery ligation (CAL) group, seven of the nine rats did not exhibit any cerebral infarct area. However, two rats in the CAL group exhibited small-sized infarct areas in the cerebral cortices, and were excluded from the study.

The 2,3,5-triphenyltetrazolium chloride (TTC) assay showed that none of the seven rats in the control group (0%) had a cerebral infarction. In the CAL group, two of the nine rats (22.2%) had small-sized infarct areas in the cerebral cortices, and were excluded from the study (Fig. 4). There were no differences in infarct volume between the control and the CAL groups (0.00 ± 0.00 vs. 0.46 ± 0.73, respectively, *p* = 0.651).

## Discussion

The results of the present study suggest that chronic cerebral hypoperfusion can contribute to AD development. Chronic cerebral hypoperfusion increased the expressions of Aβ and p-tau, which are involved in AD pathology. Furthermore, the results of the present study showed that chronic cerebral hypoperfusion selectively reduced the neuronal activity of the limbic system and impaired recognition memory. Chronic cerebral hypoperfusion is caused by small vessel disease^16^ or atherosclerosis of multiple large vessels^11^ and is known to be common in cognitively normal elderly and patients with cognitive impairment^17,18^. Thus, strategies to reduce the risk of cerebrovascular disease, including lifestyle modification, medication, rehabilitation, and surgery, can help to delay the progression of or even prevent AD dementia^5^.

It is not fully clear yet what effects chronic cerebral hypoperfusion has on the cognitive impairment. While acute cerebral hypoperfusion causes cerebral infarction through necrosis of neurons^9^, chronic cerebral hypoperfusion is expected to cause neuronal apoptosis^10^. To maintain normal neuronal activity and structural integrity, the brain requires a constant and optimal blood flow that provides glucose, the main energy source for neuronal cells^19^. In the last decade, studies have suggested that chronic cerebral hypoperfusion induces neurodegeneration via neuronal energy depletion and the generation of reactive oxygen species and proinflammatory cytokines from activated microglial cells^20,21^. One possible cause proposed for chronic cerebral hypoperfusion is cerebral small vessel disease, an atheroma of parent arteries or perforating arterioles. On brain MRI, white matter lesions are commonly seen in older people and are considered to be caused by cerebral small vessel disease^16^. In addition to cerebral small vessel disease, chronic cerebral hypoperfusion can be caused by atherosclerosis of multiple large vessels with age^11,22^. Studies have reported that normal ageing decreases the cerebral blood flow by about 20% at the age of 60 years as compared to the age of 20 years;^23^ any additional factors causing further lowering of cerebral perfusion may damage or kill vulnerable neurons^24^. In the present study, the common carotid arteries (CCA) were ligated bilaterally in Wistar rats for an animal model of chronic cerebral hypoperfusion. In accordance with the results of previous studies^25^, the CBF was decreased to about 35% of the baseline values immediately after the bilateral CCA ligation and remained at about 60% between 14 and 28 days post-CCA ligation. Permanent CCA bilateral occlusion in rodents provides a reliable model for the cognitive and histopathological studies of chronic cerebral hypoperfusion^26^. Chronic global cerebral hypoperfusion can be suitably achieved in rats as their complete circle of Willis allows a constant blood supply to the forebrain even after bilateral CCA occlusion^25^. Although in the present study small-sized infarct areas were noted after bilateral CCA ligation in the cerebral cortices of some rats, no infarct area was observed in the limbic system in any of the rats, and there was no difference in infarct volumes between the control and the CAL group. Thus, it is considered that the effects of chronic cerebral hypoperfusion on the AD pathology were adequately evaluated using this bilateral CCA ligation model in rats.

The present study revealed that chronic cerebral hypoperfusion can aggravate the AD pathology in rats. These findings are consistent with previous animal and human studies. A study using Wistar rats with bilateral CCA occlusion reported that neuroinflammation with astroglial and microglial activation as well as amyloid pathology were enhanced in the cortex, thalamus, and hippocampus^27^. Another study with mice overexpressing a mutant form of the human APP revealed that bilateral CAA surgery increased the Aβ amount in the extracellular-enriched soluble brain fraction and further impaired the learning ability by a synergistic interaction with the APP gene mutations^28^. A longitudinal study with rats showed an overexpression of the β-secretase gene on the second day and an overexpression of the APP gene on the seventh and thirtieth day after global cerebral ischemia^12^. The immunohistochemical and Western blot analyses for Aβ_42_ and Aβ_40_ may render a more specific evaluation of AD pathology. Previous studies have shown that Aβ_42_ is more fibrillogenic and toxic than the other Aβs, and intraneuronal Aβ_42_, but not Aβ_40_, is accumulated with AD pathology^29^. A study with triple transgenic AD mice revealed that chronic hypoxia increased the levels of Aβ_42_ but not Aβ_40_ without the increase in hypoxia-inducible factor 1^30^. Also, a recent study using an APP/presenilin 1 mice model and immunohistochemical analysis reported that chronic cerebral hypoperfusion caused by bilateral CCA ligation accelerated Aβ aggregation by facilitating the polymerization of high-molecular weight Aβ species^31^. A human study using MRI, cerebrospinal fluid analysis, and F-18 flutemetamol PET also revealed that lower Aβ_38_ and Aβ_40_ levels in the cerebrospinal fluid and increased uptake of F-18 flutemetamol in the cortex were associated with white matter lesions caused by small vessel disease^32^. Using MRI and F-18 florbetaben PET, our study group revealed recently that small vessel disease is associated with cerebral Aβ burden in patients with cognitive impairment^14^. In accordance with previous publications, the present study showed that the expression levels of Aβ in the frontal cortex and hippocampus, as well as the expression of p-tau in the temporal cortex, were increased at 12 weeks after bilateral CAA ligation. However, the expression of Aβ in the temporal cortex and the expression levels of p-tau in the frontal cortex and hippocampus were not changed significantly. These discrepancies between the Aβ and p-tau expression in different cerebral regions could be explained by the different pattern of cerebral Aβ and p-tau deposition according to the AD progression. It is known that Aβ deposition in the frontal and parietal cortices appears to be the early stage in the development of AD, whereas p-tau aggregates precede Aβ deposition in the temporal cortex^33^.

Medial temporal lobe atrophy, especially hippocampal atrophy, is the most important marker for the diagnosis of AD with MRI; atrophy in this region is related to episodic memory impairment. Volume loss and decreased glucose metabolism of the hippocampus is consistently reported across a wide variety of MR and F-18 FDG PET imaging studies. Significant volume losses in AD patients in the CA1 region and the entorhinal cortex, which correlated with impaired memory performance, were noted in a 7-T MRI study using volumetric analyses of hippocampal subdivisions^34^. Moreover, an F-18 FDG PET study with whole-brain modulated maps revealed significant hypometabolism in the bilateral hippocampus^35^. Another recent study using 7-T MRI and high-resolution F-18 FDG PET reported a significantly decreased glucose metabolism in the middle and posterior CA2/3 regions of the hippocampus in patients with early-stage AD^36^. In agreement with previous studies in AD patients, the present study showed a selectively decreased glucose metabolism in the amygdala, entorhinal cortex, and hippocampus but no abnormal glucose metabolism in the bilateral cerebral cortices at 12 weeks after bilateral CAA ligation. This finding supports the evidence that chronic cerebral hypoperfusion can selectively reduce the neuronal activity of the hippocampus and impair memory functions, as observed in AD. The selective decrease in hippocampal glucose metabolism is considered to be due to the vulnerability of the hippocampus to ischemic insults^37^. It has been postulated that the glucose metabolism of the cerebral cortex gradually decreases secondary to the disruption of the cortical-hippocampal connectivity in advanced stages of AD due to a functional impairment of the limbic system including the amygdala, entorhinal cortex, and hippocampus caused by chronic cerebral hypoperfusion^38,39^. In addition to the selectively decreased glucose metabolism of the limbic system, the novel object recognition test in the present study revealed that chronic cerebral hypoperfusion could impair short-term and long-term recognition memories. This finding is in accordance with the results of a previous study with Wistar rats that reported significant cognitive impairment in both the reference and working spatial memories, as well as in the non-spatial memory evaluated by a novel object recognition test after bilateral CCA occlusion surgery^40^.

The current study has some limitations. First, we performed bilateral CAA ligation surgery to induce chronic cerebral hypoperfusion in an animal model. Under clinical conditions, chronic cerebral hypoperfusion is not caused by CAA ligation. Animal models of diabetes with high-fat diet or type-I interferon injection can cause small vessel disease or multivessel atherosclerosis resulting in chronic cerebral hypoperfusion^41,42^. Studies using these animal models to identify cerebrovascular disease and confirm the occurrence of chronic cerebral hypoperfusion would be more appropriate to clarify the AD pathophysiology. Second, the effect of decreased cerebral perfusion on F-18 FDG uptake of the brain was not assessed, because we did not perform a dynamic F-18 FDG PET study. However, although the SUVR does not fully reflect the true metabolic rate of glucose, it is the most widely used quantification index in F-18 FDG brain PET imaging and could attenuate the effect of decreased cerebral perfusion on F-18 FDG uptake, because it is the ratio of the radioactivity concentration of the target and reference regions^43^. Also, because not only SUVR_cbl_ but also SUVR_WB_ calculated by setting the whole brain, where cerebral perfusion was supplied with bilateral CCA, as the reference tissue were decreased in specific regions associated with AD, the effect of decreased cerebral perfusion is considered to be minimal on our results. Further studies involving measurements of glucose metabolic rate and using animal models that better simulate cerebrovascular diseases are needed to clarify the association between chronic cerebral hypoperfusion and AD development.

## Conclusion

Chronic cerebral hypoperfusion increased the expression levels of Aβ and p-tau and selectively reduced the neuronal activity of the hippocampus in rats. These results suggest that chronic cerebral hypoperfusion can induce AD pathology. Chronic cerebral hypoperfusion may play a significant role in the development of AD. Further studies are needed to elucidate the mechanisms by which chronic cerebral hypoperfusion aggravates AD pathology.

## Materials and Methods

### Animals

Eight-week-old male Wistar rats (250–300 g body weight) were acquired from Central Lab Animal Inc. (Korea). They were kept in standard cages at a temperature of 22–24 °C with 12-h light/12-h dark cycles (08:00 lights on) and controlled humidity (55–60%). Food and water were freely accessible. All experiments in this study were performed in accordance with the guidelines for animal research from the National Institutes of Health and were approved by the Institutional Animal Care and Use Committee at Keimyung University School of Medicine.

### Bilateral common carotid artery ligation surgery

To develop the rat model of chronic cerebral hypoperfusion, the bilateral CCA was doubly ligated in 22 Wistar rats (CAL group), whereas 22 sham-operated rats were exposed to the same procedures without carotid artery ligation (control group). Rats were anaesthetized using 4.0% isoflurane in N_2_O/O_2_ (70:30) and maintained with 2.0% isoflurane in N_2_O/O_2_ (70:30). The core temperature was maintained between 37.0 °C and 38.0 °C throughout the entire procedure. A short incision was carefully performed to facilitate the separation of the bilateral CCA from the surrounding tissues. Polylysine-coated nylon was tightened around the CCA stump. In the control group, only anesthesia and vascular dissection were performed. For postsurgical care, rats were placed into separate cages and provided with freely available food and water.

### Western blot analysis

The expression of Aβ, p-tau, and APP was evaluated at 12 weeks after bilateral CAA ligation using western blots. The extracted brain tissue (right frontal cortex, temporal cortex, and hippocampus) from the seven rats in each experimental group was homogenized with T-PERTM Tissue Protein Extraction Reagent (78510; Thermo Fisher Scientific, USA) combined with proteinase inhibitor cocktail tablet 1 (cOmplete Mini, EDTA-free; Roche Applied Science, Germany) and PhosSTOP EASY(Roche Applied Science, Germany) and then incubated at 4 °C for 30 min. Samples were then centrifuged at 4 °C for 15 min. The amount of protein (10 μg) was estimated using a bicinchoninic acid assay protein assay (Pierce, Thermo Fisher Scientific, USA). Proteins separated using 10% sodium dodecyl sulfate-polyacrylamide gel electrophoresis were transferred to nitrocellulose membranes, and immunoreactive bands were visualized using a chemiluminescent reagent (SuperSignal West Femto Maximum Sensitivity Substrate, Thermo Fisher Scientific, USA). The signals of the bands were quantified with the ImageQuant software using a FUSIONSOLO5 (KOREA BIOMICS). The following antibodies were used: anti-Aβ (ab216436, Abcam, Cambridge, MA), anti-hyperphosphorylated tau (ab151559, Abcam, Cambridge, MA), anti-APP (ab32136, Abcam, Cambridge, MA), anti-GAPDH (21189, Cell Signaling Technology, Danvers, MA), anti-mouse IgG HRP-linked antibody (Santa Cruz Biotechnology, Santa Cruz, CA), and anti-rabbit IgG HRP-linked antibody (Santa Cruz Biotechnology, Santa Cruz, CA).

### F-18 FDG PET

For the evaluation of the cerebral glucose metabolism, eight rats of each group (CAL and control) underwent an F-18 FDG PET at 12 weeks after bilateral CCA ligation using the Triumph II PET/CT system (Lab-PET8; Gamma Medica-Ideas, Waukesha, WI, USA). Rats were kept fasting for 12 h before the PET scan. They were anaesthetized using 2.0% isoflurane in N_2_O/O_2_ (70:30), and approximately 37 MBq of F-18 FDG were injected via the tail vein. One hour after the injection of F-18 FDG, they were scanned using PET for 20 min. The whole-brain images of the rats were obtained, and the acquired data were assumed to represent cerebral glucose metabolism.

For quantification of the cerebral glucose metabolism in a spatiotemporal manner, a volume-of-interest analysis was performed for each scan using the PMOD software package (PMOD Technologies, Ltd., Zürich, Switzerland) in conjunction with the W. Schiffer rat brain template and atlas, as previously described^44^. PMOD was used to transform each of the rat brain PET datasets to the appropriate space, and the W. Schiffer VOI brain atlas was automatically applied to measure the F-18 FDG uptake, in standard uptake units, within defined subregions of the rat brain. The W. Schiffer brain VOI atlas was used in an iterative fashion with the standard brain model to further optimize the fusion of the experimental data. Standardized F-18 FDG uptake values were obtained from the W. Schiffer rat brain VOIs. The regional SUVR_cbl_ and SUVR_WB_ were calculated by dividing the standardized F-18 FDG uptake value for the individual target region by that for the bilateral cerebellum and whole brain, respectively.

### Novel object recognition test

The novel object recognition test was performed in eight rats of each group (CAL and control), as previously described^45,46^. This test was carried out in the open field arena, at 28 days after the surgery. The open field arena (60 cm × 60 cm × 45 cm) made of polyvinylchloride was used. Its walls and floor were colored gray. The short term memory test consisted of a single acquisition session (sampling phase), followed by a retention test 2 hours later. Two types of objects were used, different in color, shape, and material. For acquisition, each rat was placed in the middle of the arena containing two identical objects (objects A_1_ and A_2_), and was left free to explore these for a total of 1 min. The animal was then placed back in the home cage for 2 h. Object recognition was tested in a 5 min session, during which one object used during the acquisition phase was replaced by a novel object (object B). The nature and the spatial position of the objects were counterbalanced within each group in order to avoid any bias due to a preference that rats may have for a given object or its position in the arena. Object exploration was later scored using video recordings of each trial by an experimenter who was blinded to the group assignment of the rats during testing and during off-line data analysis. Exploration of an object was defined as directing the nose to the object at a distance of less than 2 cm or touching it with the nose; turning around or sitting on the object was not considered as exploration. A recognition index calculated for each animal was expressed by the ratio T_B_ /(T_A_ + T_B_). [T_A_ = time spent exploring the familiar object A; T_B_ = time spent exploring the novel object B]. Between trials the objects were washed with 10% ethanol solution. In a long-term memory test given 24 h after training, the same rats explored the field for 5 min in the presence of familiar object A and a novel object C.

### Laser-Doppler flowmetry

The CBF was measured in eight rats of each group (CAL and control) using laser-Doppler flowmetry (OMEGA FLOW FLO-C1 BV, OMEGAWAVE, Tokyo, Japan), as previously reported^47^. Under deep anesthesia with 4.0% isoflurance in N_2_O/O_2_(70:30), the right skull was reflected. A laser-Doppler flowmetry probe was fixed perpendicularly to the skull at 1 mm posterior and 2.5 mm lateral to the bregma using dental resin. The CBF recordings were obtained just before (baseline) and at 2 hours and 3, 7, 14, and 28 days after the surgery. The CBF values were expressed as a percentage of the baseline value.

### TTC assay

To evaluate the cerebral infarction, a TTC assay was performed at 1 day after bilateral CCA ligation. Seven rats in the control group and nine rats in the CAL group were sacrificed. Their brains were removed and sliced into five coronal sections with a thickness of 2 mm. These sections were immersed in prewarmed 2% TTC (Sigma) in saline for 15 min and then fixed in 4% paraformaldehyde overnight. White parts of the brain indicated cerebral infarct areas, whereas normal tissue regions were stained red by TTC. The infarct area of each brain was measured in a blinded manner using ImageJ (National Institutes of Health, Bethesda, Maryland, USA). The infarct volume was calculated using Swanson’s method^48^. The areas of non-infarcted gray matter were measured using the ImageJ. The areas were each summed over the number of sections evaluated and the respective volumes were calculated by multiplying each sum by the distance between sections. The infarct volumes of the lesioned structures were expressed as a percentage of the volume of the structures in the control hemispheres. The rats showing cerebral infarction with the TTC assay were excluded from the Western blot and F-18 FDG PET analyses.

### Statistical analysis

Differences in Aβ, p-tau, and APP expression levels, and regional SUVR, recognition index, CBF, and infarct volume between the CAL and control groups were evaluated using the Mann-Whitney U test. The *p*-values were corrected for multiple comparisons using a false discovery rate correction. A *p*-value of <0.05 was considered statistically significant. Data for all study variables are expressed as means ± standard deviations.

## Supplementary information

Supplementary Table 1

## Data Availability

The datasets generated and/or analyzed during the current study are available from the corresponding author on reasonable request.
