# Supplementary material for: The effect of chronic cerebral hypoperfusion on the pathology of Alzheimer's disease: A positron emission tomography study in rats
Source: Sci Rep. 2019 Oct 1;9:14102. doi: 10.1038/s41598-019-50681-4 (PMC6773854; doi:10.1038/s41598-019-50681-4)
Supplement: Supplementary file 1 — Supplementary Table 1 [file 41598_2019_50681_MOESM1_ESM.docx]

**Supplementary Information**

**The effect of chronic cerebral hypoperfusion on the pathology of Alzheimer's disease: A positron emission tomography study in rats**

Jae-Hyung Park^1^, Jeong-Ho Hong^2^, Sang**-**Woo Lee^3^, [Hyun Dong](https://www.google.co.kr/url?sa=t&rct=j&q=&esrc=s&source=web&cd=1&ved=2ahUKEwjerPyc2ozdAhVbdt4KHXPhBwsQFjAAegQIABAB&url=https%3A%2F%2Fwww.researchgate.net%2Fprofile%2FJi_Hyun_Dong&usg=AOvVaw3hbIil_4qmfMpru4Lo05Hq) Ji^3^, Jung-Ah Jung^1^, Kyung-Wha Yoon^4^, Jung-In Lee^4^, Kyoung Sook Won^4^, Bong-Il Song^4^, & Hae Won Kim^4,*^

^1^Department of Physiology, School of Medicine, Keimyung University, Daegu, Republic of Korea

^2^Department of Neurology, School of Medicine, Keimyung University, Daegu, Republic of Korea

^3^Department of Nuclear Medicine, School of Medicine, Kyungpook National University, Daegu, Republic of Korea

^4^Department of Nuclear Medicine, School of Medicine, Keimyung University, Daegu, Republic of Korea

^*^ E-mail: hwkim.nm@gmail.com

**Supplementary Table 1.** Comparisons of regional standardized uptake value ratios^*^ between the control and bilateral common carotid artery ligation (CAL) groups

| Regions | Side | Control group | CAL group | *p*-value |
| --- | --- | --- | --- | --- |
| Accumbens | Lt | 1.04 (0.09) | 1.02 (0.06) | 0.600 |
|  | Rt | 1.05 (0.05) | 1.08 (0.09) | 1.000 |
| Amygdala | Lt | 0.92 (0.06) | 0.85 (0.03) | 0.021 |
|  | Rt | 0.89 (0.06) | 0.87 (0.03) | 0.462 |
| Auditory cortex | Lt | 0.92 (0.04) | 0.92 (0.06) | 0.753 |
|  | Rt | 0.88 (0.03) | 0.90 (0.04) | 0.294 |
| Cingulate cortex | Lt | 1.04 (0.13) | 1.08 (0.05) | 0.600 |
|  | Rt | 1.03 (0.16) | 1.03 (0.07) | 0.753 |
| Entorhinal cortex | Lt | 0.94 (0.06) | 0.89 (0.05) | 0.036 |
|  | Rt | 0.93 (0.04) | 0.89 (0.05) | 0.093 |
| Frontal association cortex | Lt | 0.93 (0.09) | 0.93 (0.08) | 0.834 |
|  | Rt | 0.89 (0.03) | 0.96 (0.07) | 0.016 |
| Insular cortex | Lt | 1.02 (0.08) | 1.02 (0.05) | 0.916 |
|  | Rt | 0.99 (0.05) | 1.02 (0.04) | 0.345 |
| Medial prefrontal cortex | Lt | 1.15 (0.13) | 1.17 (0.13) | 0.916 |
|  | Rt | 1.16 (0.10) | 1.15 (0.11) | 0.834 |
| Motor cortex | Lt | 0.92 (0.09) | 0.95 (0.04) | 0.834 |
|  | Rt | 0.91 (0.08) | 0.92 (0.02) | 0.834 |
| Orbitofrontal cortex | Lt | 1.05 (0.08) | 1.05 (0.07) | 0.834 |
|  | Rt | 1.06 (0.04) | 1.07 (0.09) | 0.600 |
| Para cortex | Lt | 0.83 (0.07) | 0.85 (0.06) | 0.401 |
|  | Rt | 0.83 (0.09) | 0.83 (0.08) | 0.834 |
| Retrosplenial cortex | Lt | 0.89 (0.06) | 0.9 (0.05) | 0.753 |
|  | Rt | 0.91 (0.08) | 0.9 (0.06) | 0.674 |
| Somatosensory cortex | Lt | 0.92 (0.06) | 0.95 (0.02) | 0.172 |
|  | Rt | 0.91 (0.07) | 0.94 (0.05) | 0.345 |
| Visual cortex | Lt | 0.84 (0.07) | 0.85 (0.06) | 0.834 |
|  | Rt | 0.84 (0.07) | 0.83 (0.05) | 0.600 |
| Hippocampus anterodorsal | Lt | 1.04 (0.05) | 1.01 (0.05) | 0.208 |
|  | Rt | 1.05 (0.05) | 1.02 (0.05) | 0.172 |
| Hippocampus posterior | Lt | 1.01 (0.09) | 0.92 (0.07) | 0.043 |
|  | Rt | 1.00 (0.09) | 0.95 (0.03) | 0.229 |

^*^ Regional standardized uptake value ratio (SUVR_WB_) was calculated by dividing the standardized uptake value for each regional VOI by the standardized uptake value for the whole brain as a reference region. All values are presented as mean (standard deviation).
